# Supplementary material for: Targeting AURKA-CDC25C axis to induce synthetic lethality in ARID1A-deficient colorectal cancer cells
Source: Nat Commun. 2018 Aug 10;9:3212. doi: 10.1038/s41467-018-05694-4 (PMC6086874; doi:10.1038/s41467-018-05694-4)
Supplement: Supplementary file 1 — Supplementary Information [file 41467_2018_5694_MOESM1_ESM.pdf]

Supplementary Information

Targeting AURKA-CDC25C axis to induce synthetic lethality in ARID1A-deficient colorectal cancer cells

Wu et al.

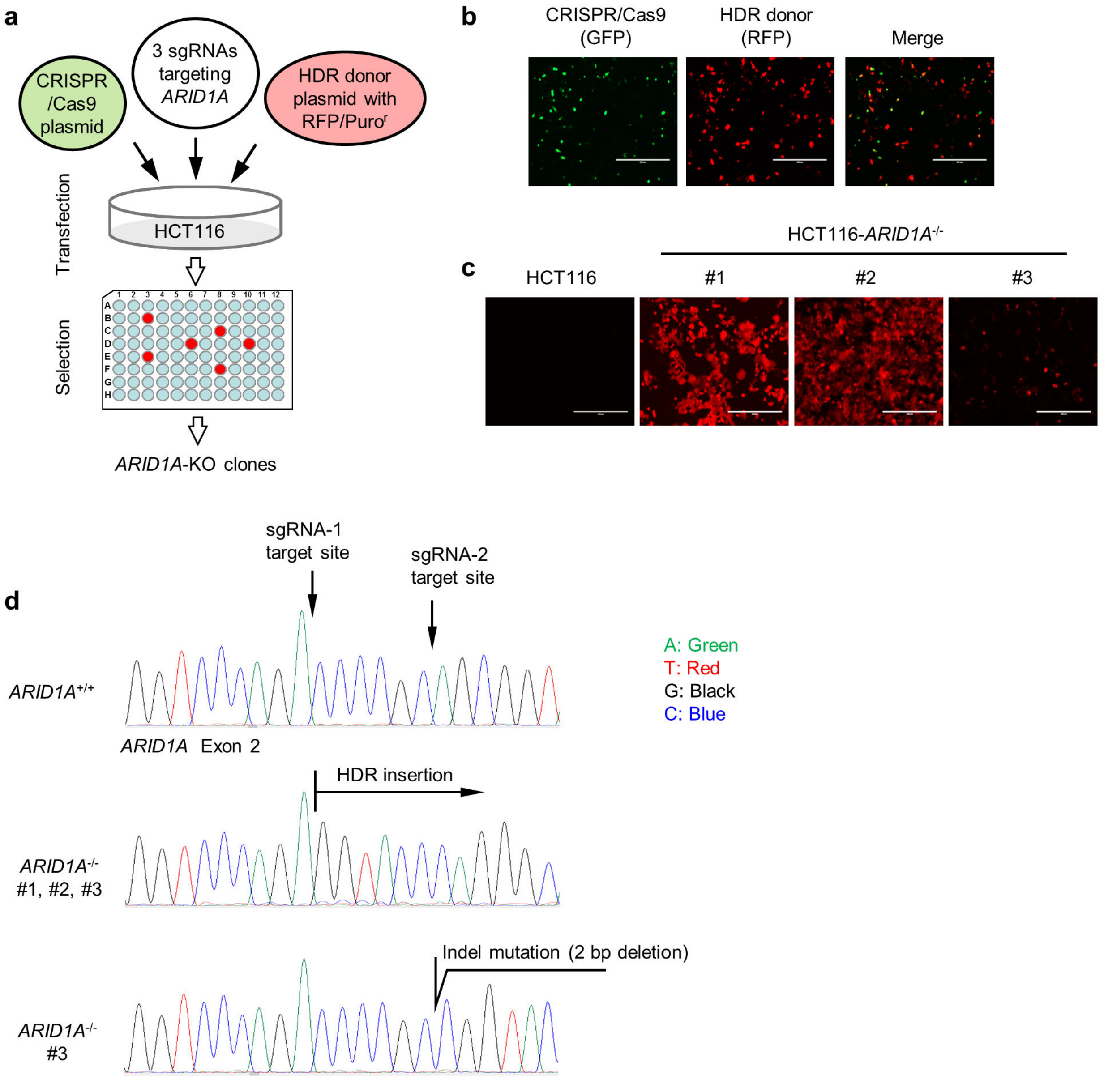

Supplementary Figure 1. Generation of *ARID1A* knockout (KO) HCT116 cells. a, HCT116 cells were transfected with CRISPR/Cas9 plasmid containing green fluorescence protein (GFP), 3 sgRNAs targeting *ARID1A* gene and HDR donor plasmid containing red fluorescence protein (RFP) and puromycin resistant gene ($Puro^r$). After 48 h of transfection, puromycin was added. After 72 h, the cells were trypsinized and plated in 96-well plates at approximately 0.5-1 cell per well for clone selection. Single clones were picked up after they form colonies. **b**, Transfection efficiency was assessed with GFP (CRISPR/Cas9) and RFP (HDR donor plasmid). Co-transfectants are shown as yellow in the merged image. Scale bars, 400 μm. **c**, After 2 weeks of selection and clone isolation, *ARID1A*$^{-/-}$ clones have red fluorescence only. Scale bars, 400 μm. **d**, Sequencing analysis of the sgRNA target sites on *ARID1A* exon 2 in HCT116-*ARID1A*$^{+/+}$ and HCT116-*ARID1A*$^{-/-}$ clones. *ARID1A*$^{-/-}$ #1 and #2 have the HDR insertion at the sgRNA-1 target site. *ARID1A*$^{-/-}$ #3 is a heterozygote mutant that has the HDR insertion at the sgRNA-1 target site and an indel mutation (2 bp deletion) at the sgRNA-2 target site. No mutation was observed in sgRNA-3 target site on *ARID1A* exon 4 in the three *ARID1A*$^{-/-}$ clones.

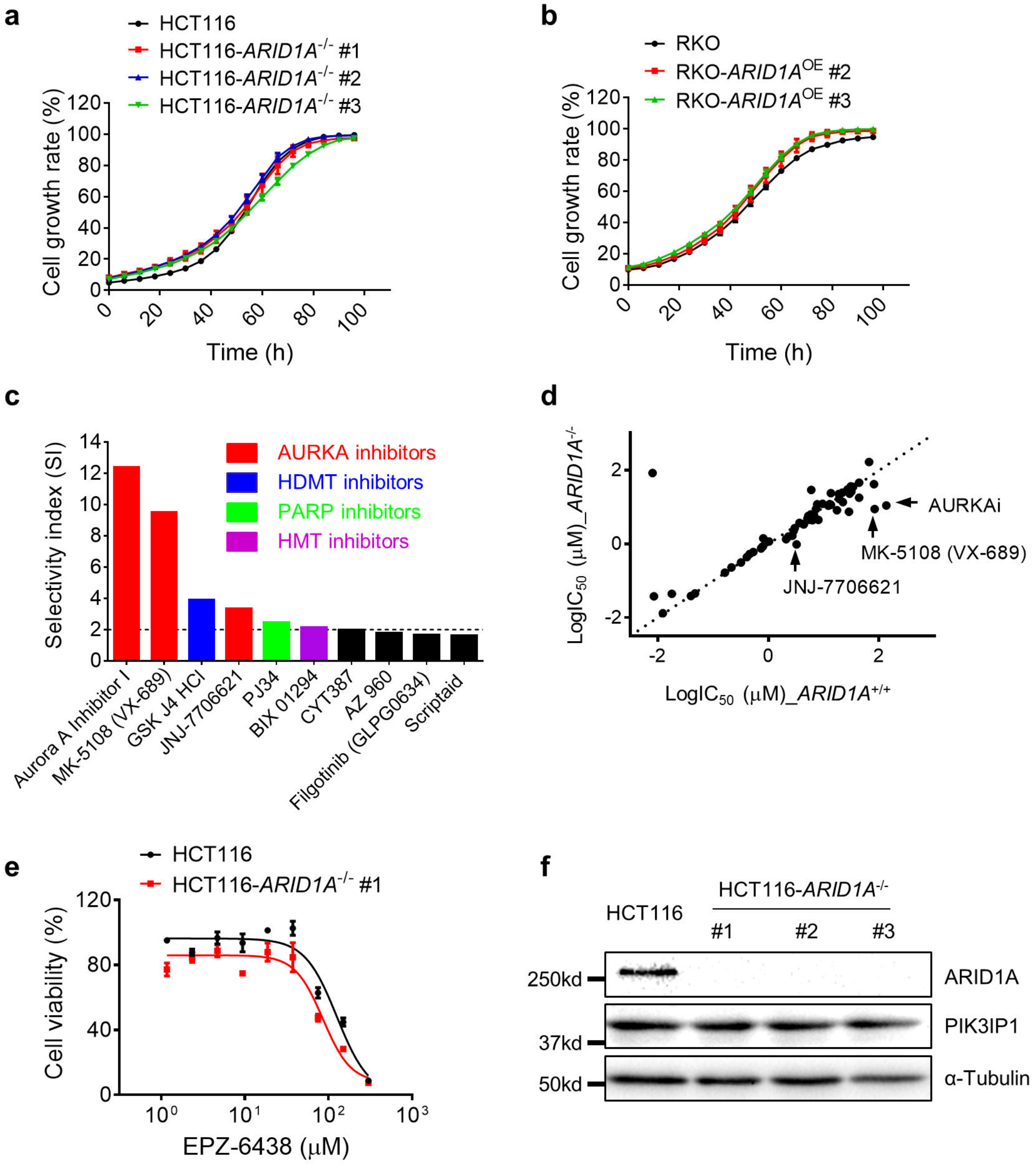

Supplementary Figure 2. The growth rates of *ARID1A*-isogenic colorectal cancer cells and synthetic lethality screening. *ARID1A*-isogenic HCT116 (**a**) and RKO (**b**) cell lines were grown in a 96-well ImageLock Microplate until confluent and assessed for real-time growth rate with IncuCyte-ZOOM. Error bars represent s.d. No significant difference was observed in in vitro growth rate between $ARID1A^{+/+}$ and $ARID1A^{-/-}$ cells. **c**, Selectivity index (SI) of the synthetic lethality candidates for ARID1A. SI = $IC_{50}^{ARID1A(+/+)}/IC_{50}^{ARID1A(-/-)}$. Among all the epigenetics compounds tested, top 10 candidates are shown in the graph and drugs with SI>2 are indicated. **d**, AURKA inhibitors identified from the screening. **e**, Dose response curves of *ARID1A*-isogenic HCT116 cell lines with EPZ-6438 (EZH2 inhibitor) treatment for 96 h. Error bars represent s.d. **f**, Immunoblot analysis of ARID1A and PIK3IP1 expression in *ARID1A*-isogenic HCT116 cell lines.

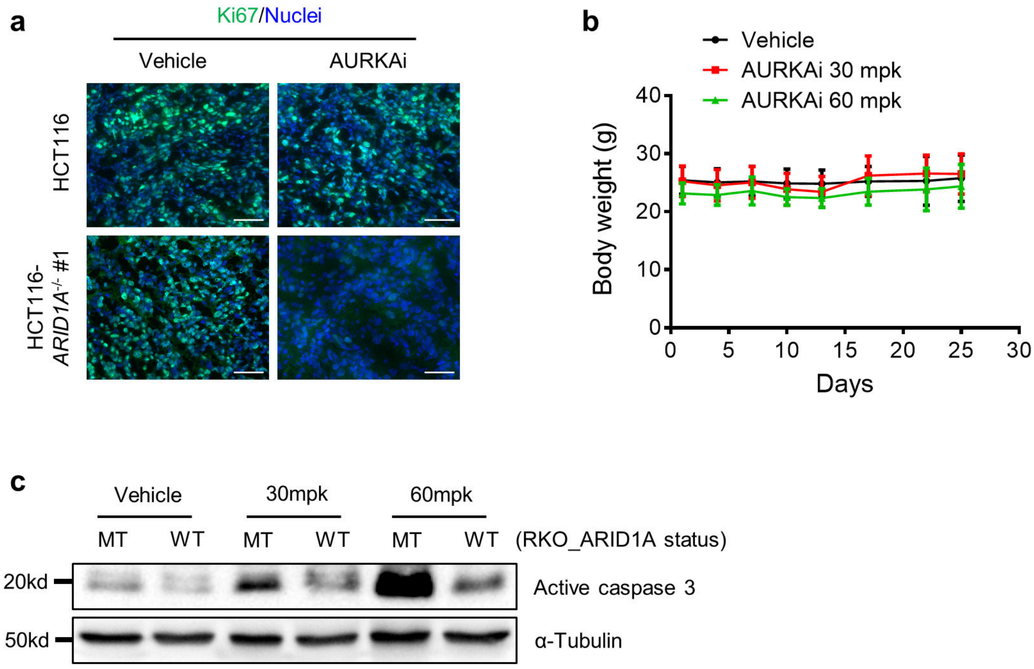

Supplementary Figure 3. *ARID1A*-isogenic CRC mouse xenograft model. a, Immunofluorescence staining of tumor tissues isolated from mice with a cell proliferation marker, Ki67 (green) and a nuclear counter staining with Hoechst33342 (blue). *ARID1A*$^{-/-}$ tumor show significant lower level of Ki67 staining upon AURKAi treatment. Scale bars, 50 μm. **b**, Mouse body weight measurement of mice treated with AURKAi. Error bars represent s.d. A daily i.p. injection of AURKAi (30 and 60 mg kg^{-1} (mpk)) did not significantly affect mice body weights. **c**, Induction of apoptosis in ARID1A-deficient RKO tumor by AURKAi treatment. Tumor tissues were lysed and subjected to immunoblot analysis of active casepase-3.

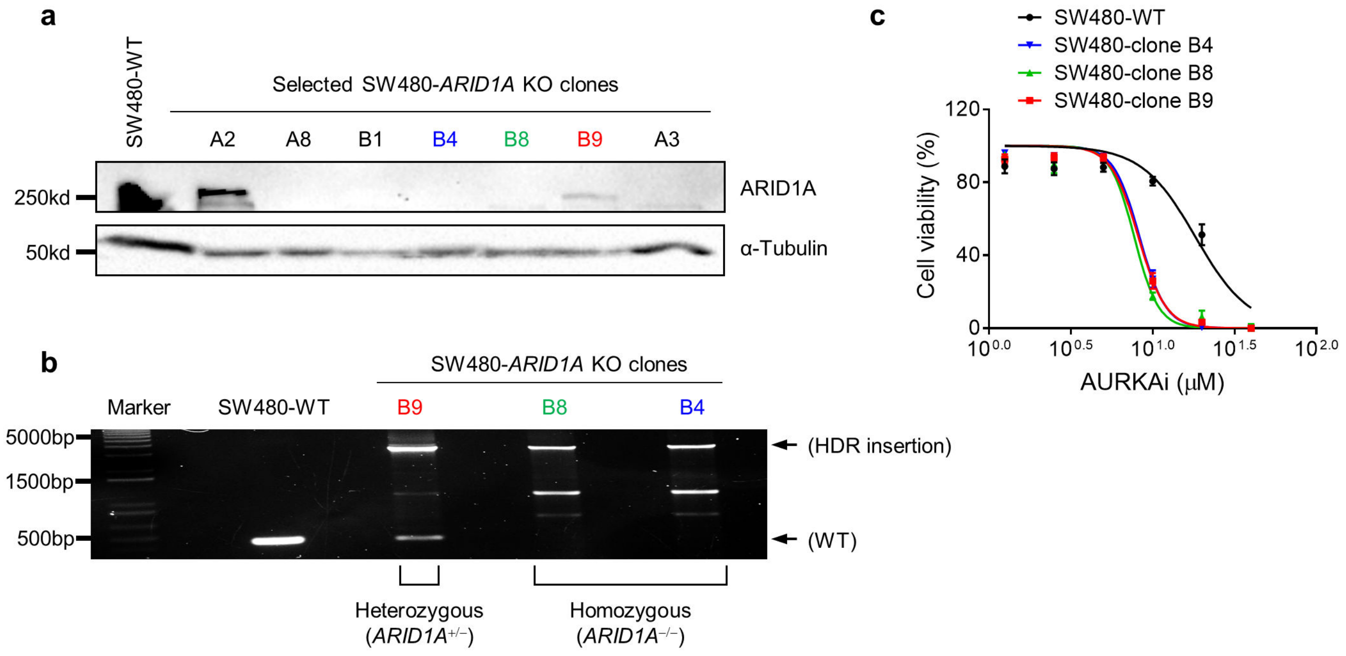

Supplementary Figure 4. Generation of SW480 *ARID1A*-KO cells and validation of ARID1A-AURKA synthetic lethality. SW480 colorectal cancer cells harboring wildtype *ARID1A* was transfected with CRISPR/Cas9 plasmid, sgRNAs targeting *ARID1A* genomic locus, and homologous-directed repair (HDR) donor plasmid containing puromycin-resistance and red fluorescence protein (RFP) genes to generate *ARID1A* KO cell lines. The RFP fluorescent cells were picked up and the KO clones were further selected with puromycin. **a**, Immunoblot analysis of ARID1A expression in SW480 parental and puromycin-selected *ARID1A* KO clones. Clones B4 and B8 have no detectable ARID1A expression, while clone B9 has barely detectable ARID1A expression. **b**, The three clones (B4, B8, and B9) were selected for genomic PCR analysis with primer pairs specific for sgRNA target site within *ARID1A* locus. Clone B9 has both *ARID1A* wildtype amplicon (WT) and an amplicon with HDR sequences (HDR insertion), suggesting an *ARID1A*-heterozygous KO clone. B8 and B4 clones only have HDR insertion amplicon, suggesting *ARID1A*-homozygous KO clones. **c**, Dose response curves of SW480 *ARID1A*-isogenic cell lines with AURKAi treatment. Error bars represent s.d. Both heterozygous and homozygous *ARID1A* KO clones show significantly increased sensitivity to AURKAi treatment.

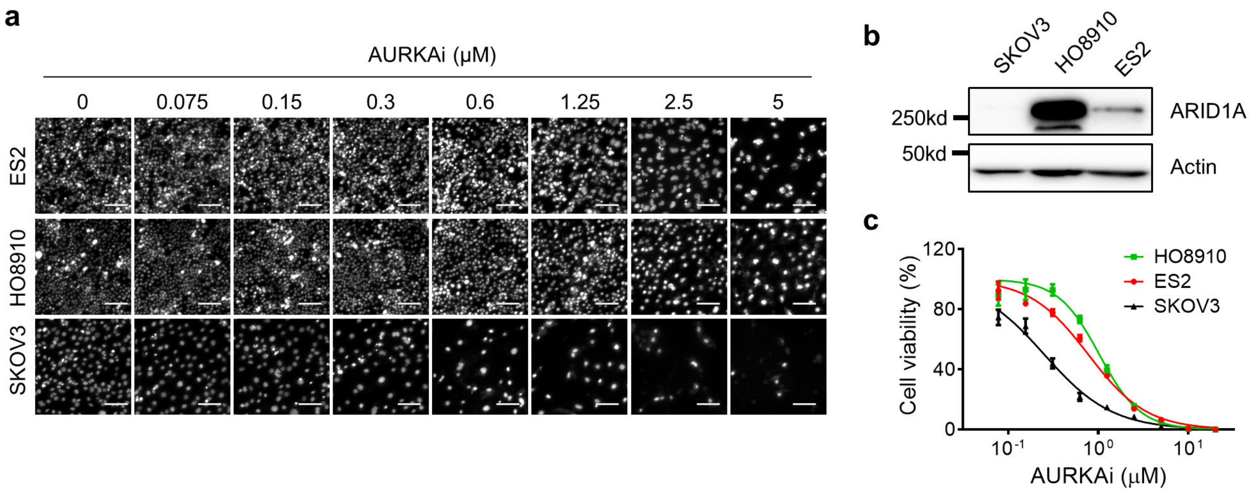

Supplementary Figure 5. Validation of ARID1A-AURKA synthetic lethality in ovarian cancer cell lines. **a**, Three ovarian cancer cell lines with different ARID1A status, ES2 (*ARID1A*-WT), HO8910 (*ARID1A*-WT), and SKOV3 (*ARID1A*-MT) were treated with AURKAi for three days and nuclei were stained with Hoechst 33342. Scale bars, 100 μm. **b**, Western blot analysis of ARID1A status in three ovarian cancer cell lines. Actin was used as an internal control. **c**, The cell viability was measured by counting the number of cell nuclei with Image J software. Error bars represent s.d.

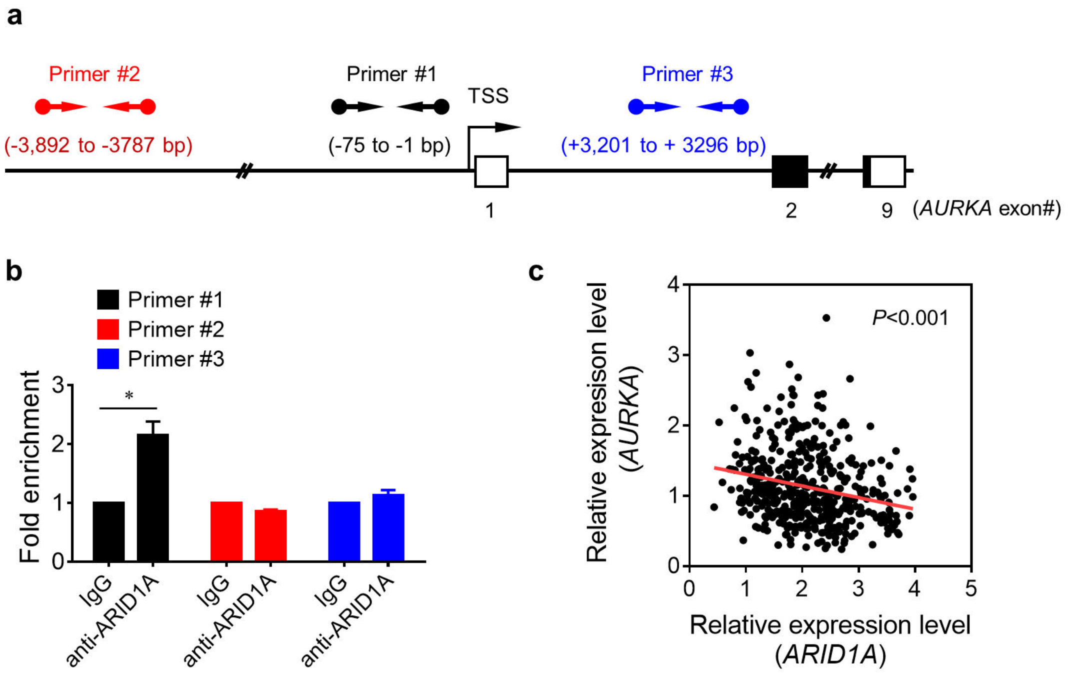

Supplementary Figure 6. Negative regulation of AURKA expression by ARID1A. **a**, Target regions of primer pairs used for chromatin immunoprecipitation (ChIP) of *AURKA* promoter with an anti-ARID1A antibody. TSS denotes the transcription start site. **b**, *AURKA* promoter ChIP was done with an anti-ARID1A antibody and three indicated primer pairs in HCT116 cells. Error bars represent s.d. ∗*P* <0.05, One sample *t*-test. **c**, The transcriptome profiling data of 440 colorectal cancer patients' samples were obtained from The Cancer Genome Atlas (TCGA). The relative mRNA expression levels of *ARID1A* and *AURKA* in each sample were analyzed. Spearman correlation analysis was used to assess statistical significance.

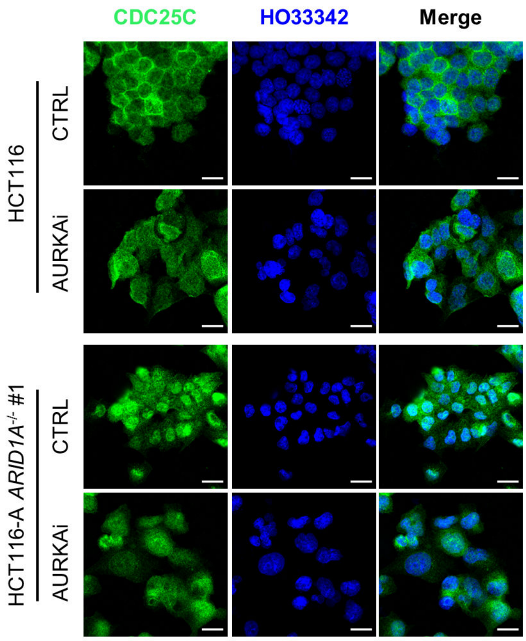

Supplementary Figure 7. Inhibition of CDC25C nuclear localization by AURKAi. *ARID1A*-isogenic HCT116 cells were treated with or without AURKAi and analyzed for immunofluorescence of CDC25C (green). Nuclei were stained with Hoechst33342 (HO33342). Note the significant nuclear localization of CDC25C in *ARID1A*-KO cells and its reversal by AURKAi treatment. Scale bars, 20 μm.

Figure 1c

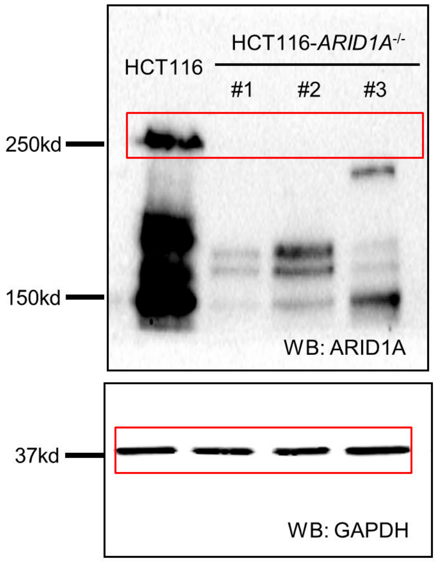

Figure 2c

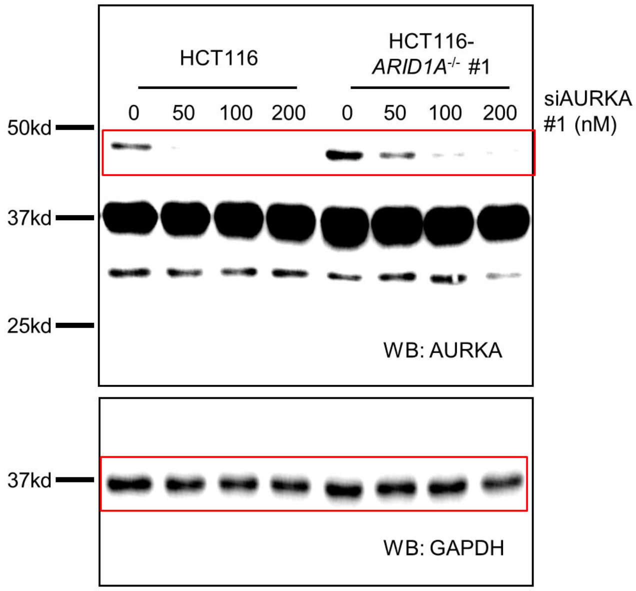

Figure 2f

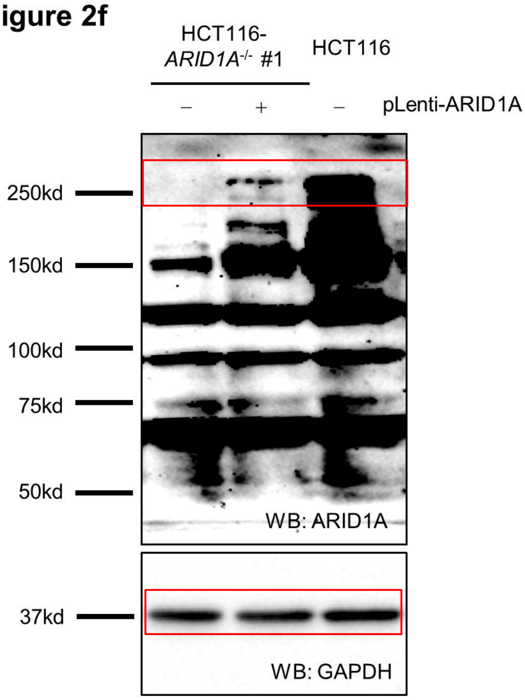

Figure 3a

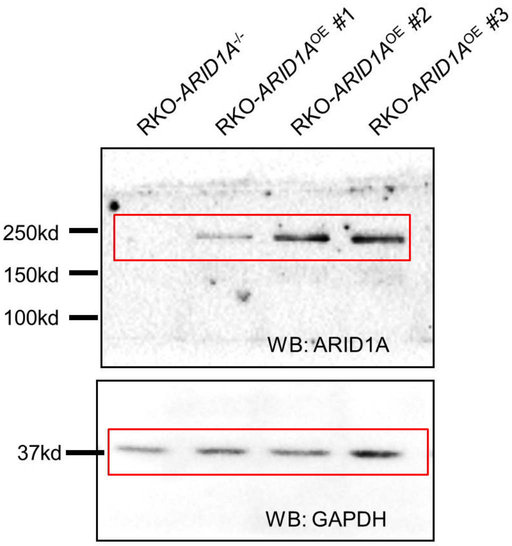

Supplementary Figure 8. Original Western blots shown in Figures 1-3. Each figure corresponds to the Western blots in the indicated Figure number.

Figure 4h

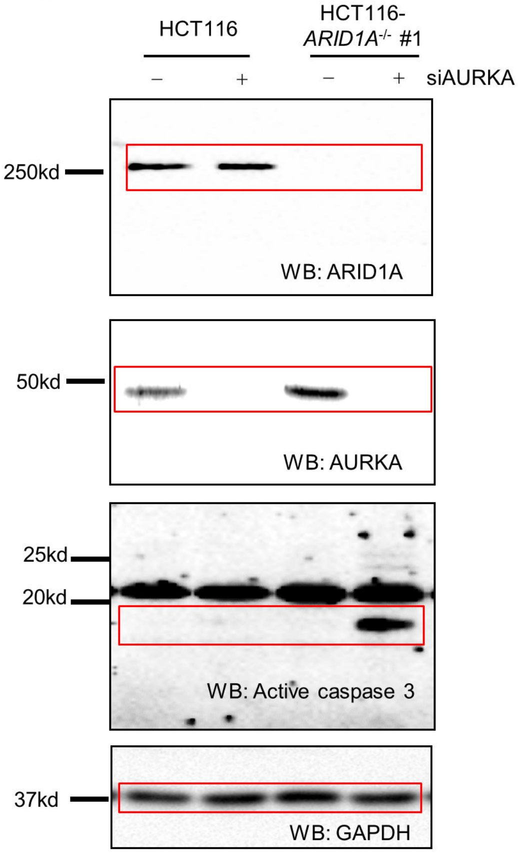

Figure 4i

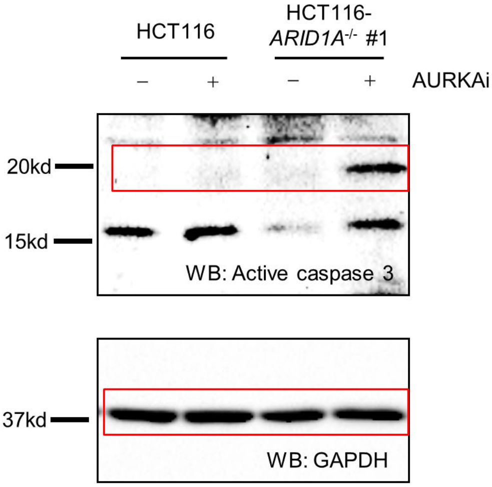

Figure 5a

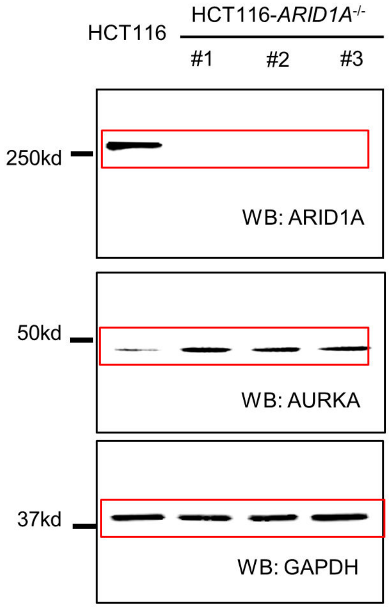

Figure 5b

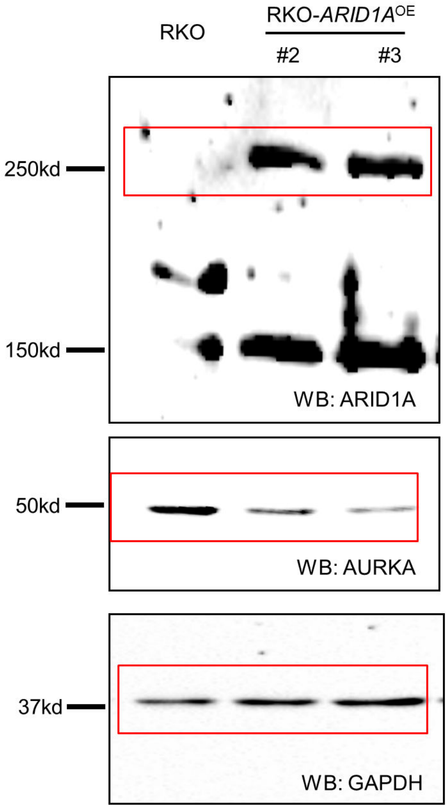

Supplementary Figure 9. Original Western blots shown in Figures 4-5. Each figure corresponds to the Western blots in the indicated Figure number.

Figure 5c

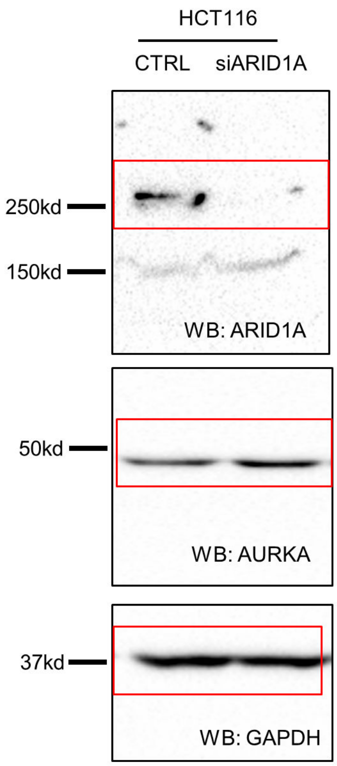

Figure 5d

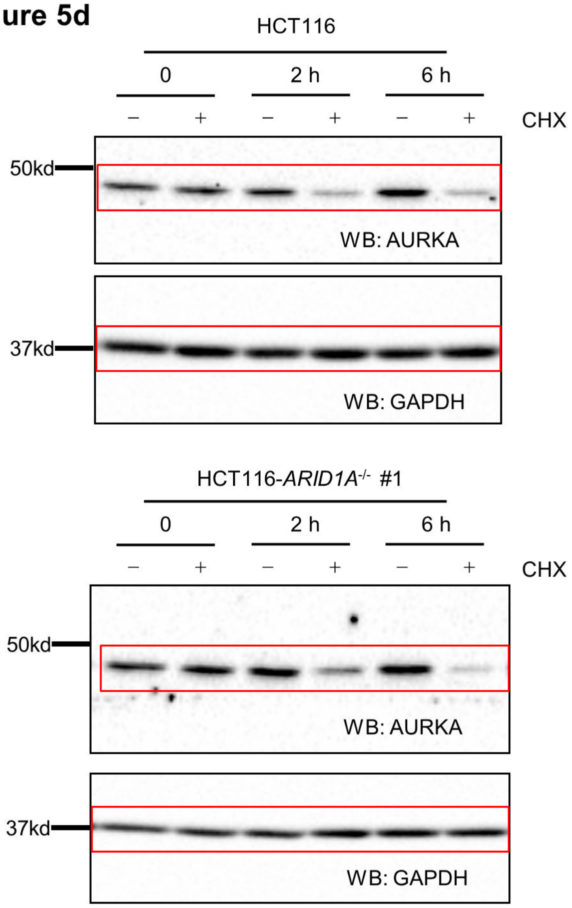

Figure 5m

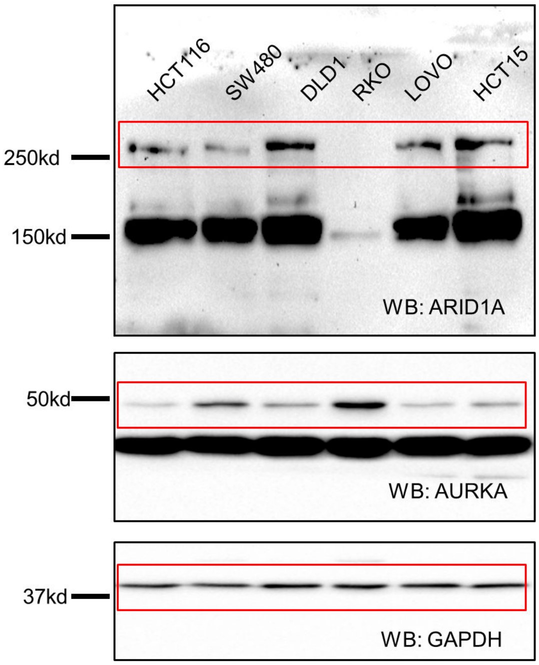

Supplementary Figure 10. Original Western blots shown in Figure 5. Each figure corresponds to the Western blots in the indicated Figure number.

Figure 6a

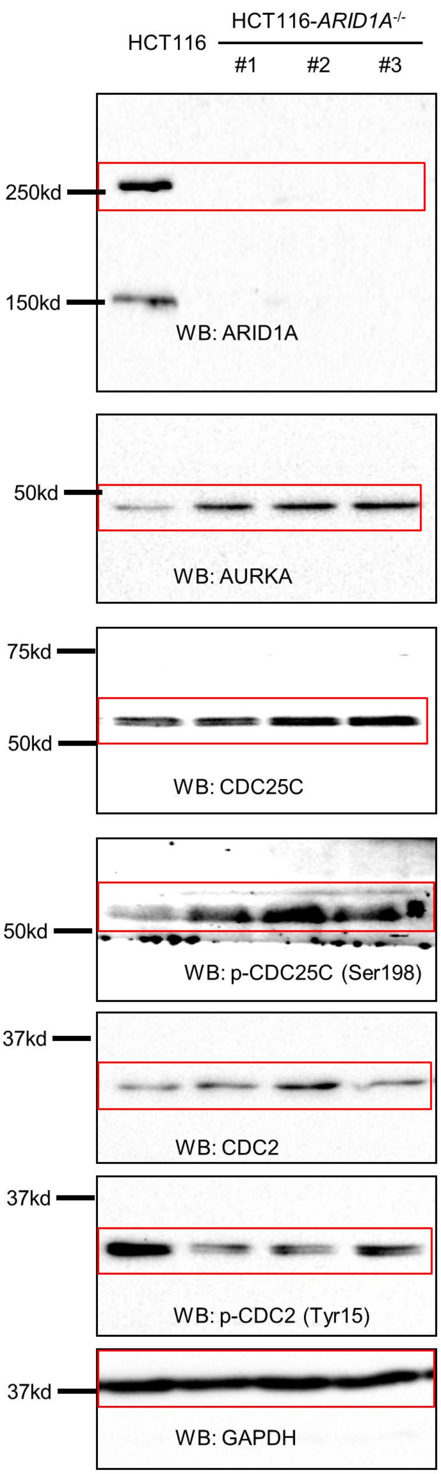

Figure 6b

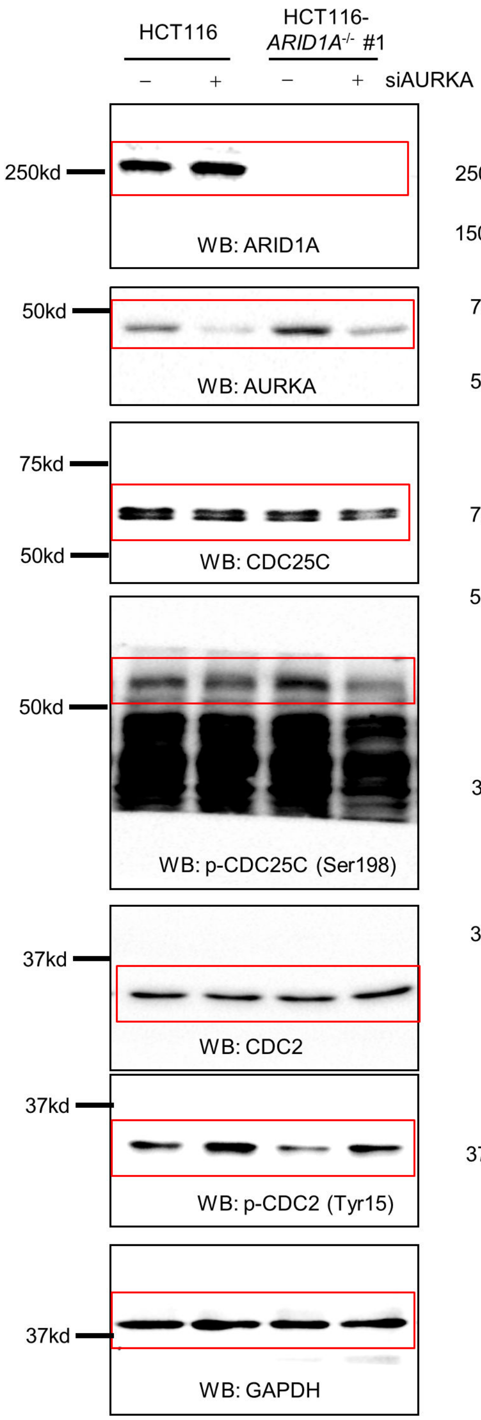

Figure 6c

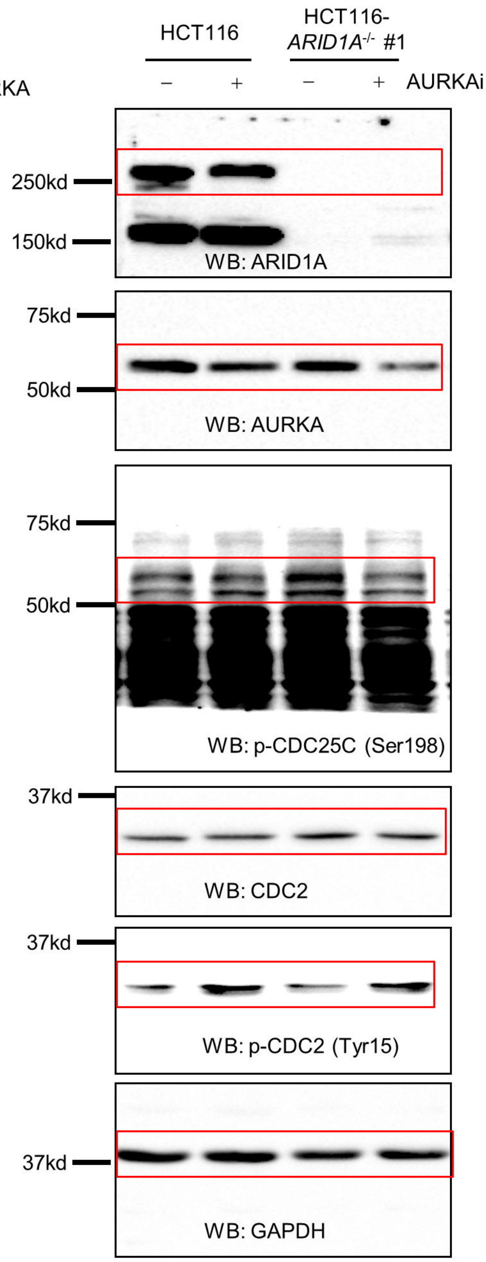

Supplementary Figure 11. Original Western blots shown in Figure 6. Each figure corresponds to the Western blots in the indicated Figure number.

Supplementary Figure 2f

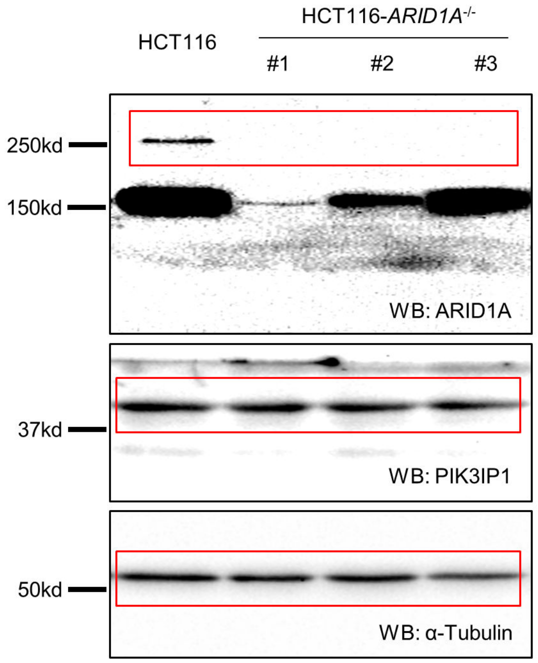

Supplementary Figure 3c

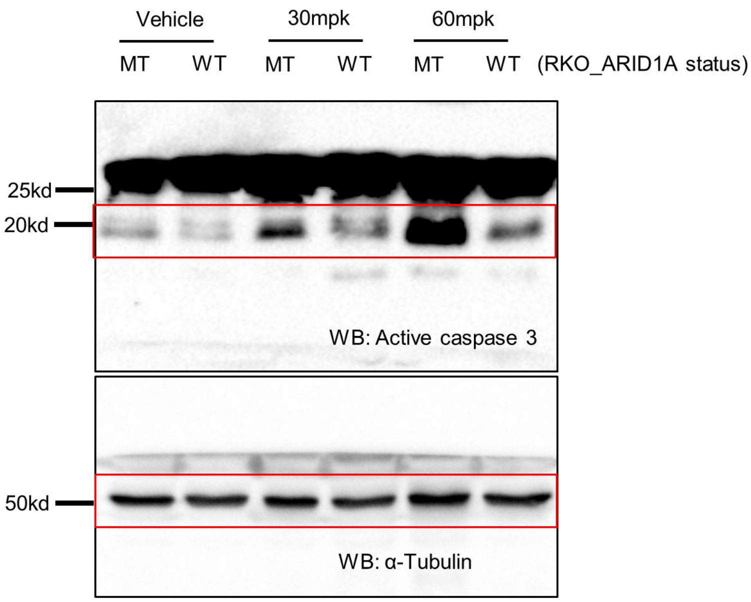

Supplementary Figure 4a

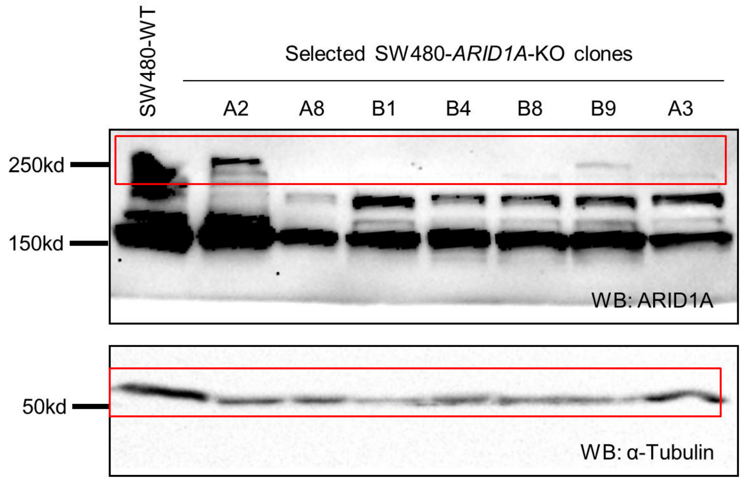

Supplementary Figure 5b

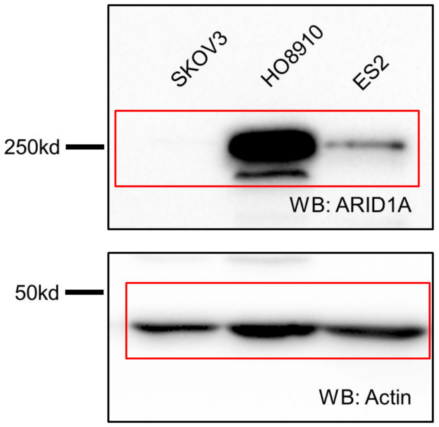

Supplementary Figure 12. Original Western blots shown in Supplementary Figures 2-5. Each figure corresponds to the Western blots in the indicated Figure number.
